# Choroid Plexus MRI Features and Cognitive Outcomes in Multiple Sclerosis: A Scoping Review

**DOI:** 10.3390/brainsci16080829

**Published:** 2026-08-04

**Authors:** Weronika Galus, Patrycja Romaniszyn-Kania, Aleksandra Urantówka, Hanna Zielonka, Katarzyna Zawiślak-Fornagiel, Julia Wyszomirska, Urszula Kłosińska, Oskar Bożek, Daniel Ledwoń, Aleksandra Tuszy, Andrzej W. Mitas, Joanna Siuda

**Affiliations:** 1 Department of Neurology, Faculty of Medical Sciences in Katowice, Medical University of Silesia, 40-752 Katowice, Poland; kzawislak-fornagiel@sum.edu.pl (K.Z.-F.); jsiuda@sum.edu.pl (J.S.); 2 Faculty of Biomedical Engineering, Silesian University of Technology, 41-800 Zabrze, Poland; patrycja.romaniszyn-kania@polsl.pl (P.R.-K.); au312831@student.polsl.pl (A.U.); hz312613@student.polsl.pl (H.Z.); dledwon@polsl.pl (D.L.); aleksandra.tuszy@polsl.pl (A.T.); andrzej.mitas@polsl.pl (A.W.M.); 3 Department of Psychology, Faculty of Health Sciences in Katowice, Medical University of Silesia, 40-752 Katowice, Poland; julia.wyszomirska@gmail.com; 4 Faculty of Psychology, SWPS University of Social Sciences and Humanities, 53-238 Wroclaw, Poland; uklosinska@swps.edu.pl; 5 Department of Radiology and Nuclear Medicine, Faculty of Medical Sciences in Katowice, Medical University of Silesia, 40-752 Katowice, Poland; oskarbozek@sum.edu.pl

**Keywords:** multiple sclerosis, choroid plexus, cognitive impairment, cognitive function, scoping review, neuroinflammation, magnetic resonance imaging, choroid plexus volume, symbol digit modalities test

## Abstract

**Highlights:**

**What are the main findings?**

- Larger choroid plexus volume was associated in several studies with poorer cognition in MS, particularly processing speed and visuospatial memory.
- Longitudinal evidence did not show consistent predictive value for choroid plexus volume; one study suggested prognostic value of the CP T1/T2 ratio.

**What are the implications of the main findings?**

- Choroid plexus volume and microstructural measures are promising MRI research markers, but not yet established clinical biomarkers.
- Harmonized acquisition and segmentation and adequately powered longitudinal, multidomain studies are needed.

**Abstract:**

Background/Objectives: Multiple sclerosis (MS) is frequently accompanied by cognitive impairment, yet the neurobiological mechanisms underlying cognitive heterogeneity remain incompletely understood. The choroid plexus (CP), a blood–CSF barrier structure involved in cerebrospinal fluid production and neuroimmune signaling, has recently emerged as a potential MRI marker of inflammatory and neurodegenerative activity in MS. This scoping review mapped evidence on associations between CP features and cognitive functions in adults with MS. Methods: A broad search of PubMed, Scopus, Web of Science Core Collection, and the Cochrane Library identified 1163 records; after deduplication, screening, and full-text assessment, seven studies were included. Results: CP volume or normalized CP volume was assessed in all seven studies, and one study additionally examined the CP T1/T2 ratio. The Symbol Digit Modalities Test was used in six studies, while five applied multidomain cognitive assessment. Larger CP volume was associated in several studies with poorer baseline information-processing speed, visuospatial memory, or multidomain cognition, but longitudinal findings did not show consistent predictive value for CP volume. One study reported that a higher CP T1/T2 ratio predicted faster visuospatial-memory decline. Conclusions: CP-related measures may reflect broader neuroinflammatory and neurodegenerative processes relevant to cognition in MS, but the evidence remains limited and methodologically heterogeneous. Standardized acquisition and segmentation, harmonized cognitive assessment, and adequately powered longitudinal studies are needed to establish their independent and predictive value.

## 1. Introduction

Multiple sclerosis (MS) is a chronic immune-mediated disease of the central nervous system characterized by inflammatory demyelination, axonal injury, and progressive neurodegeneration. Cognitive impairment is one of the most frequent and clinically meaningful manifestations of MS and may involve information processing speed, attention, memory, and executive functions. These deficits can substantially affect daily functioning, occupational performance, treatment adherence, and quality of life [1,2]. Although focal inflammatory lesions contribute to cognitive dysfunction, they do not fully explain its heterogeneity. Increasing evidence indicates that chronic, compartmentalized inflammation, often conceptualized as “smouldering MS”, contributes to tissue injury, brain atrophy, disability accumulation, and possibly cognitive decline [3,4]. Chronic active lesions, characterized by persistent inflammatory activity at the margins of longstanding demyelinated lesions, represent one manifestation of this process; a subset can be identified in vivo as paramagnetic rim lesions, also termed iron rim lesions, on susceptibility-sensitive MRI [5]. Compartmentalized inflammation may also involve CNS border compartments that mediate immune–CNS communication, including the cerebral microvascular blood–brain barrier, the meninges and leptomeningeal spaces, and the choroid plexus/blood–CSF barrier [4,6,7,8].

The choroid plexus (CP) is a highly vascularized structure located within the ventricular system. It is the main source of cerebrospinal fluid (CSF), forms the blood–CSF barrier, and contributes to immune surveillance, CSF composition, metabolic exchange, and CNS homeostasis [6]. Because of its position at the interface between the peripheral immune system and CNS fluid spaces, the CP is increasingly regarded as a neuroimmune gateway that may participate in immune cell trafficking and inflammatory signaling [7]. In MS, CP alterations may therefore reflect or contribute to blood–CSF barrier dysfunction, intrathecal inflammation, and compartmentalized neuroinflammatory activity [8].

Neuroimaging studies suggest that CP enlargement and other CP-derived MRI measures may occur early in MS and may be associated with inflammatory activity, lesion burden, tissue damage, brain atrophy, and disease progression [9,10,11]. At the same time, advances in manual, automated, and deep learning-based CP segmentation have made CP assessment increasingly feasible in larger clinical and research cohorts [12,13]. However, the relationship between CP features and cognitive function in MS remains insufficiently mapped. Existing studies differ in clinical populations, MRI protocols, CP segmentation methods, cognitive tests, and covariate adjustment. A recent narrative review by De Meo et al. provided a broad overview of cognition in MS, including its role in detecting disease progression, biological and MRI correlates, and treatment-related considerations [14]. A 2025 systematic review and meta-analysis of 27 studies reported higher normalized CP volume in patients with MS than in healthy controls and associations with disability and global MRI measures [15]. However, neither publication provided a dedicated study-level synthesis of CP features in relation to individual cognitive tests and domains, definitions of impairment, or longitudinal cognitive outcomes.

The present scoping review therefore addresses a distinct clinical and methodological question by focusing specifically on CP–cognition evidence. The objective of this scoping review is to analyze and map the available literature on associations between choroid plexus features and cognitive functions in adults with MS.

## 2. Materials and Methods

### 2.1. Design and Reporting Standards

This review was conducted as a scoping review in accordance with Chapter 11 of the JBI Manual for Evidence Synthesis [16] and reported according to the PRISMA Extension for Scoping Reviews (PRISMA-ScR) [17]. This scoping review is registered in the Open Science Framework (OSF): https://osf.io/3upm5/ (accessed on 6 June 2026).

### 2.2. Eligibility Criteria

Eligibility criteria followed the Population–Concept–Context framework. The population included adults with clinically diagnosed MS, regardless of phenotype or care setting. Pediatric, animal, in vitro, and non-MS studies without separate MS data were excluded. The concept comprised CP-specific features, including volume, enlargement, morphology, segmentation, and other MRI-derived CP measures. Studies assessing only ventricles, CSF, or non-CP brain structures were excluded unless CP-specific data were reported. Eligible outcomes included cognitive function, cognitive impairment, neuropsychological performance, or cognitive test results in people with MS. Reviews, meta-analyses, protocols, editorials, letters, case reports, theoretical articles, and conference abstracts without full text were excluded. Detailed criteria are provided in Supplementary Table S1.

### 2.3. Information Sources and Search Strategy

PubMed, Scopus, Web of Science Core Collection, and the Cochrane Library were searched during April and May 2026, from database inception to the date of each search. The strategy combined two core concepts: multiple sclerosis and choroid plexus. A mandatory cognition-related search block was not used to maximize sensitivity, as some studies assessed cognition without highlighting it in titles or abstracts. No publication date, language, age or study-design restrictions were applied. Reference lists of included studies and relevant reviews were screened manually. The complete database-specific search strategies are presented in Supplementary Table S2.

### 2.4. Study Selection

All records were imported into Rayyan, where duplicates were removed before screening. Two reviewers independently screened titles and abstracts, followed by full-text assessment of potentially eligible articles. Disagreements were resolved by discussion, with consultation of a third reviewer when needed. Reasons for exclusion at the full-text stage were recorded.

### 2.5. Data Charting, Extraction and Synthesis

Data were extracted using a standardized charting form developed for this review and piloted before final extraction. Extracted items included study design, population characteristics, MS phenotype and clinical data, CP assessment method, cognitive outcomes, neuroimaging findings, statistical approach, covariates, limitations, and reported research gaps. Missing or unassessed items were recorded as “Not reported” or “Not assessed”, respectively.

Findings were summarized descriptively according to study design, MS phenotype, CP metric, MRI method, cognitive domain, cognitive test, and direction of association between CP measures and cognitive outcomes. Due to heterogeneity in study designs, imaging protocols, segmentation methods, and cognitive assessments, no meta-analysis was performed. Given the small number of eligible studies and substantial heterogeneity in CP measures, cognitive outcomes, study designs, and statistical reporting, quantitative pooling was not considered appropriate. As this review was designed as a scoping review, its primary aim was to map and characterize the available evidence rather than to estimate a pooled effect. Detailed MRI acquisition characteristics were summarized in Supplementary Table S3, whereas statistical methods, covariate adjustment, reliability, study limitations, and JBI appraisal were reported in Supplementary Table S4.

### 2.6. Critical Appraisal

Methodological quality was appraised using appropriate JBI critical appraisal tools [16]. Analytical cross-sectional studies were assessed with the JBI checklist for analytical cross-sectional studies, while studies including longitudinal follow-up were assessed using the JBI checklist for cohort studies. Appraisal focused on participant selection, CP measurement, cognitive outcome assessment, confounding, follow-up where applicable, and statistical analysis. No study was excluded based on appraisal results; findings were used only to contextualize the evidence.

## 3. Results

### 3.1. Search Results and Study Selection

**Figure 1:**
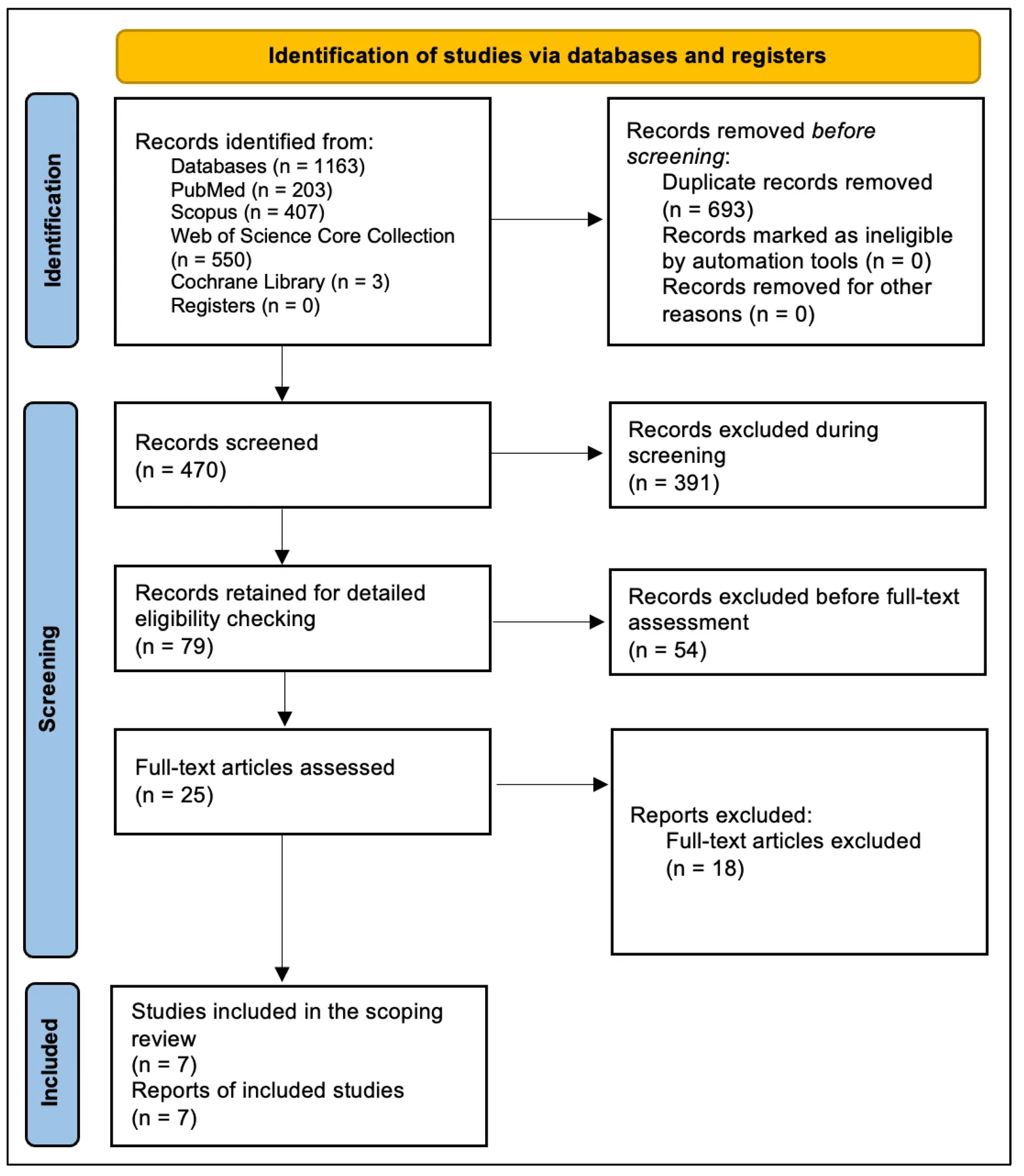
PRISMA flow diagram of the literature search and study selection process.

This literature search was conducted across four databases using the following concepts: multiple sclerosis and choroid plexus. This approach identified a total of 1163 records. After duplicate removal, it was determined that only 470 articles were unique. Using the inclusion and exclusion criteria detailed in Supplementary Materials, Table S1, 391 records were excluded during the title and abstract screening, leaving only 79 records for detailed eligibility assessment. Fifty-four records were excluded before fill-test assessment, and 25 full-text articles were assessed. Eighteen full-text articles were excluded, and seven studies met all eligibility criteria and were included in this review. The outcomes of all study selection stages are presented in Figure 1.

### 3.2. Characteristics of Included Studies

The seven included studies were published between 2023 and 2026 and were conducted in China, Italy, Poland and the United Kingdom. They included X. Wang et al. 2023 [18], Kania et al. 2024 [19], Preziosa et al. 2024 [20], X. Wang et al. 2024 [21], De Meo et al. 2025 [22], Z. Wang et al. 2025 [23], and Galus et al. 2026 [24]. Four studies were primarily cross-sectional [18,20,23,24], whereas three included longitudinal follow-ups [19,21,22]. The study populations focused mainly on relapsing-remitting MS. One study included both relapsing-remitting and progressive MS phenotypes [20], and one recruited RRMS and PPMS patients but analyzed the main cognitive findings primarily in RRMS [24]. The two studies including progressive MS did not provide adequately powered, prespecified phenotype-stratified analyses of CP–cognition associations [20,24].

CP volume or normalized CP volume was assessed in all seven studies, while De Meo et al. additionally evaluated the CP T1/T2 ratio as a microstructural measure [22]. MRI protocols primarily included 3D T1-weighted imaging, FLAIR, T2-weighted imaging, diffusion MRI, and susceptibility-based methods such as SWI or QSM. Most cohorts were examined at 3 T, one study used a 1.5 T system [24], and the external multicenter dataset used by X. Wang et al. applied non-standardized T1-weighted protocols [21]. CP segmentation ranged from manual delineation to FreeSurfer-based volumetry, automated in-house segmentation with visual inspection and correction, Gaussian-mixture refinement, and deep learning-based 3D UX-Net segmentation [18,19,20,21,22,23,24]. Detailed scanner characteristics, MRI acquisition parameters, and CP segmentation, normalization, and validation procedures for all included studies are presented in Supplementary Table S3.

Cognitive assessment also varied substantially. The Symbol Digit Modalities Test (SDMT), reflecting information processing speed, was used in six of the seven studies. Kania et al. used a different broad neuropsychological battery [19]. Five studies applied multidomain cognitive assessment covering verbal memory, visuospatial memory, attention, executive function, processing speed, and/or verbal fluency [19,20,22,23,24]. Three studies used domain-based classifications, with multidomain impairment defined by abnormal performance in at least two cognitive domains [20,23,24]. One study used a MoCA cut-off score below 26 [18], and one defined cognitive worsening as a decrease in SDMT raw score of at least 4 points or 10% [21]. Kania et al. and De Meo et al. analyzed longitudinal cognitive performance without applying a single binary definition of cognitive impairment [19,22].

Several cross-sectional analyses associated larger CP volume with poorer cognitive performance or cognitive impairment, although the strength and independence of these associations varied. In X. Wang et al. 2023, associations with cognition were attenuated after adjustment for lesion burden or brain volumes [18], whereas Preziosa et al. and Z. Wang et al. reported associations with multidomain cognitive impairment [20,23]. X. Wang et al. 2024 observed an inverse association between baseline nCPV and SDMT performance [21], De Meo et al. found an independent association between higher nCPV and lower BVMT-R scores [22], and Galus et al. reported modest associations with verbal-fluency measures [24]. Longitudinal findings were mixed. Baseline nCPV did not predict two-year SDMT worsening in X. Wang et al. [21]; baseline CP volumes were not associated with follow-up semantic fluency or Word Comprehension Test performance in Kania et al. [19]; and baseline nCPV did not predict change in BICAMS outcomes in De Meo et al. [22]. However, De Meo et al. found that a higher baseline CP T1/T2 ratio predicted faster decline in visuospatial memory [22]. Study and population characteristics are presented in Table 1, while CP measures, cognitive assessment, and principal findings are summarized in Table 2.

### 3.3. Critical Appraisal of Included Studies

Critical appraisal was conducted using the appropriate JBI tools [16]. Overall, two studies were judged as having low methodological concerns [20,23], and five studies were judged as having some concerns [18,19,21,22,24]. No study was excluded based on critical appraisal. The most frequent methodological limitations were cross-sectional design, limited or selected samples, attrition in the longitudinal cohorts, heterogeneity in MRI acquisition and CP segmentation methods, incomplete reporting of CP-specific reliability or normalization, restricted cognitive assessment in some studies, and variable adjustment for potential confounders such as lesion burden, brain atrophy, ventricular volume, disease duration, disability, treatment status, depression, fatigue, and education. Detailed statistical and methodological appraisal data are presented in Supplementary Table S4.

## 4. Discussion

This scoping review identified seven studies examining associations between CP features and cognition in adults with MS. CP volume or nCPV remained the dominant MRI measure, while one study additionally assessed CP microstructure using the T1/T2 ratio [22]. Several studies associated larger CP volume with poorer contemporaneous cognitive performance or cognitive impairment, although the strength, cognitive domain, and independence of these associations varied. The most consistent findings concerned information-processing speed and visuospatial memory, with less consistent evidence for verbal fluency. Because four studies were primarily cross-sectional and the longitudinal findings were heterogeneous, the results should be interpreted as associations rather than proof that CP enlargement independently causes or predicts cognitive decline.

The SDMT was used in six of the seven studies and therefore remained the most frequently assessed cognitive measure. This is relevant because the SDMT is a validated and widely recommended measure of information-processing speed in MS cognitive assessment [25,26]. However, reliance on SDMT alone may underestimate the multidomain nature of MS-related cognitive impairment, which may also involve memory, executive functions, attention, and verbal fluency [26,27,28]. De Meo et al. found that higher normalized CP volume was independently associated with poorer baseline visuospatial memory, but not with SDMT or verbal-learning performance [22]. Baseline CP volume did not predict subsequent cognitive change in either X. Wang et al. or De Meo et al. [21,22]. Kania et al. found no association between baseline CP volume and follow-up semantic fluency or Word Comprehension Test performance, although these analyses were unadjusted and based on a small MRI sample [19]. In contrast, De Meo et al. reported that a higher baseline CP T1/T2 ratio predicted faster decline in visuospatial memory [22]. Thus, CP volume may reflect current inflammatory or neurodegenerative burden more clearly than future cognitive decline, whereas CP microstructural measures may have prognostic potential that requires independent replication.

Methodological heterogeneity limits direct comparison between studies. Acquisition protocols were not directly comparable across studies. Most cohorts were scanned at 3 T, one at 1.5 T, and an external multicenter dataset used non-standardized T1-weighted protocols [18,19,20,21,22,23,24]. Scanner hardware, sequence contrast, spatial resolution, contrast administration, lesion filling, reconstruction and segmentation also varied, while complete acquisition parameters were not always reported. These differences may affect tissue contrast, CP boundary delineation, and partial-volume effects, particularly for this small, anatomically complex structure surrounded by CSF. Therefore, between-cohort differences in CP measures cannot be attributed solely to biological variation or field strength. CP segmentation varied from manual delineation to FreeSurfer-based volumetry, automated in-house methods, Gaussian-mixture refinement, and deep learning-based 3D UX-Net segmentation [12,13,18,19,20,21,22,23,24]. Normalization and covariate adjustment were also inconsistent. This is important because the CP is a small structure located within the ventricular system, and CP volume may partly covary with ventricular enlargement, global atrophy, lesion burden, or disease severity. Future studies should therefore report segmentation reliability and consistently adjust for intracranial volume, ventricular volume, lesion burden, and regional or global brain atrophy [12,13].

Clinical heterogeneity is another important limitation. Most cohorts were restricted to RRMS and generally included patients with mild disability. Progressive MS was represented only in one mixed cohort and in a small PPMS subgroup, without adequately powered phenotype-specific CP–cognition analyses [20,24]. DMT exposure was also reported using non-equivalent classifications and differed in timing and treatment switching across cohorts [19,21,22]. These data could not be reliably harmonized and may contribute to residual confounding of associations between CP measures and cognition. Findings obtained predominantly in mildly disabled RRMS cohorts should therefore not be generalized to progressive MS.

The biological link between CP abnormalities and cognition is likely multifactorial. CP enlargement on MRI is unlikely to represent a single histopathological process. Activation of epithelial, stromal, vascular, and immune compartments may alter blood–CSF barrier homeostasis, increase cytokine, chemokine, and adhesion-molecule signaling, and facilitate immune-cell retention and trafficking at the CSF interface [6,7,8]. Experimental autoimmune encephalomyelitis studies further identify the CP as an immune-cell reservoir and potential route of entry into CNS fluid compartments [7]. Changes in the inflammatory CSF milieu could promote periventricular and meningeal inflammatory signaling and downstream microglial and astrocytic activation, potentially contributing to diffuse white- and gray-matter injury within networks supporting processing speed, attention, memory, and executive functions. However, MRI-derived CP volume does not directly measure epithelial activation, barrier permeability, immune-cell content, cytokine activity, or glial activation. These mechanisms should therefore be regarded as biologically plausible interpretations rather than demonstrated mediators of the observed CP–cognition associations.

CP volume may also not capture the full spectrum of CP pathology. De Meo et al. found that a higher CP T1/T2 ratio predicted visuospatial-memory decline, whereas CP volume did not predict longitudinal cognitive change [22]. In Z. Wang et al., the DTI-ALPS index partially mediated 27.21% of the association between CP volume and SDMT and 43.75% of the association between CP volume and delayed visuospatial memory [23]. Complementary studies outside the cognitive eligibility criteria of this review linked larger CP volume with lower DTI-ALPS indices and suggested that altered perivascular diffusion may partially mediate associations between CP enlargement and lesion burden, ventricular enlargement, and regional brain-volume loss [29,30]. A recent RRMS study without CP assessment also found that a lower ALPS index was independently associated with cognitive impairment and poorer processing speed, attention/working memory, and verbal fluency [31]. Together, these findings suggest potential interactions among CP pathology, CSF production and turnover, perivascular fluid transport, structural brain damage, and cognitive dysfunction. Nevertheless, DTI-ALPS is an indirect diffusion-based measure, and a recent comparison with intrathecal contrast-enhanced MRI demonstrated only limited correspondence with brain-wide CSF tracer dynamics [32]. Lower DTI-ALPS values should therefore not be interpreted as direct proof of glymphatic dysfunction.

At least three non-mutually exclusive interpretations of CP enlargement should therefore be considered. First, CP dysfunction could contribute to cognitive injury through altered blood–CSF barrier regulation, immune-cell trafficking, inflammatory signaling, CSF homeostasis, or perivascular fluid transport. Second, CP enlargement could be a consequence of persistent neuroinflammation, treatment-related changes, altered CSF dynamics, ventricular enlargement, or structural tissue loss elsewhere in the brain. Third, it may act primarily as a surrogate marker of cumulative disease burden, correlating with lesion load, chronic active lesions, atrophy, disability, and treatment exposure without having an independent causal role. Current evidence cannot reliably distinguish these possibilities because cross-sectional designs predominate, some associations were attenuated after adjustment for lesion burden or brain volumes, and the three longitudinal studies yielded heterogeneous findings [18,19,20,21,22,23,24].

Accordingly, CP measures should currently be considered candidate research markers rather than direct measures of CP inflammation or established clinical biomarkers. Their greatest value may lie in multimodal models integrating CP volumetry and microstructure with lesion burden, PRLs or IRLs, ventricular volume, regional and global atrophy, inflammatory biomarkers, measures of perivascular fluid transport, and cognitive trajectories. Future studies should use harmonized MRI acquisition and segmentation procedures, repeated imaging, multidomain cognitive batteries, prespecified definitions of cognitive worsening, and standardized adjustment for education, depression, fatigue, disease duration, disability, lesion burden, brain atrophy, ventricular volume, and treatment exposure [26,27,28]. Larger longitudinal cohorts should include progressive MS phenotypes and permit prespecified stratified or interaction analyses according to phenotype and treatment efficacy.

This review addresses a focused and emerging topic and used a broad search strategy based on MS and CP concepts, which helped capture studies assessing cognition even when cognitive outcomes were not highlighted in titles or abstracts. Its main limitations are the inclusion of only seven studies, four primarily cross-sectional studies and heterogeneous longitudinal designs, attrition in the longitudinal cohorts, RRMS-focused populations with generally mild disability, and substantial variation in MRI acquisition, segmentation, cognitive assessment, and covariate adjustment. In addition, CP volume remained the principal measure, while CP microstructure was assessed in only one study. The small number of studies and the lack of sufficiently comparable CP measures, cognitive outcomes, and effect estimate also precluded quantitative pooling.

Overall, larger CP volume was associated with poorer baseline cognition in several studies, particularly information-processing speed and visuospatial memory, but its independence, specificity, and predictive value remain unresolved.

## 5. Conclusions

The available evidence from seven studies suggests that CP-related MRI features, particularly CP volume, may be associated with cognitive outcomes in MS. Larger CP volume was linked in several studies to poorer baseline information-processing speed, visuospatial memory, or multidomain cognitive impairment, but longitudinal evidence did not demonstrate consistent predictive value for CP volume. One study suggested that the CP T1/T2 ratio may be associated with subsequent visuospatial-memory decline. CP measures should therefore be regarded as candidate research markers rather than direct measures of CP inflammation or established clinical biomarkers. Standardized MRI acquisition and CP segmentation, harmonized multidomain cognitive assessment, and adequately powered longitudinal studies are required to establish the independent and clinical value of CP measures.

## Figures and Tables

**Table 1:** Study and population characteristics of the included studies.

Study/Year	Country/Setting	Design	Follow-Up	Sample	MS Phenotype	Age, Years	Disease Duration, Years	EDSS	DMT Reporting
X. Wang et al., 2023 [18]	China; Chongqing Medical University	Retrospective cross-sectional MRI case–control study	None	99 RRMS + 60 age-/sex-matched HCs	RRMS only	Mean 33.62	Median 2.83	Median 1.0	None 48%; modest 32%; moderate 20%
Kania et al., 2024 [19]	Poland; Poznan University of Medical Sciences	Single-center longitudinal cohort	Median 6 y (baseline 2014–2015; follow-up 2020–2022)	41 baseline; 30 follow-up; 27 usable baseline MRIs	RRMS only	Mean 36 at baseline	Mean 6 at baseline	Median 1.0 baseline; 2.0 follow-up	All treated; treatment switching during follow-up
Preziosa et al., 2024 [20]	Italy; San Raffaele, Milan	Retrospective cross-sectional MRI case–control study	None	129 MS + 73 age-/sex-matched HCs	RRMS 94 (73%); PMS 35 (27%)	Mean 43.3	Median 9.14	Median 2.0	None 9%; first-line 57%; second-line 34%
X. Wang et al., 2024 [21]	China; internal single-center and external multicenter datasets	Retrospective segmentation-development study with cross-sectional and longitudinal analyses	2 y in Dataset 1b	Dataset 1a: 163 RRMS + 75 HCs; Dataset 1b: 53 RRMS; Dataset 2: 58 RRMS/67 scans	RRMS only	NR	NR	NR	Dataset 1b: treated 33; untreated 20
De Meo et al., 2025 [22]	United Kingdom; POINT-MS	Prospective cohort	Baseline, 6 mo, and 18 mo	422 RRMS; 276 at 6 mo; 80 at 18 mo	RRMS only	Mean 40.8	Mean 9.5	Median 2.0	High-efficacy 257; moderate/low-efficacy 165; recruited around treatment initiation
Z. Wang et al., 2025 [23]	China; Lanzhou University/Gansu imaging centers	Cross-sectional MRI case–control study with mediation and random-forest analyses	None	70 RRMS + 44 HCs	RRMS only	Median 31.0	Median 2.5	Median 2.5	Regular 67.1%; intermittent 20.0%; none 12.9%
Galus et al., 2026 [24]	Poland; Medical University of Silesia/Silesian University of Technology	Cross-sectional clinical MRI-neuropsychological study	None	79 recruited; 70 analyzed (63 RRMS, 7 PPMS)	RRMS and PPMS; principal cognitive/volumetric analyses in RRMS	NR	RRMS mean 12.0; PPMS mean 9.4	RRMS median 2.0; PPMS median 5.0	RRMS: platform 30.2%; high-efficacy 69.8%

Note: DMT categories are reported as defined in the original studies and should not be interpreted as directly comparable across cohorts. NR = not reported.

**Table 2:** Choroid plexus measures, cognitive assessment, and principal findings.

Study	CP Metric	Segmentation/Normalization	Cognitive Test/Domain	Definition of Impairment or Worsening	Main Cross-Sectional Result	Main Longitudinal Result
X. Wang et al., 2023 [18]	Lateral-ventricle CPV; normalized to TIV	Manual ITK-SNAP; third- and fourth-ventricle CP excluded	MoCA; SDMT	MoCA < 26 = CI; SDMT z-scores derived from HCs	CPV was 30% higher in RRMS than HCs (*p* < 0.001) and 20% higher in IRL-positive than IRL-negative RRMS (*p* = 0.007). CI RRMS had higher CPV (*p* = 0.011); the SDMT association was attenuated after adjustment for lesion load/brain volumes.	Not assessed
Kania et al., 2024 [19]	Right and left CP compartment volumes	FreeSurfer v5.3 aseg; CP-specific normalization and reliability NR	Broad battery; CP analyses focused on semantic fluency and WCT	No single binary CI definition for CP analyses	No CP-specific cross-sectional cognition result reported	Baseline right/left CP volumes were not associated with follow-up semantic fluency (r = −0.068 and 0.103) or WCT (r = −0.091 and −0.159); all *p* ≥ 0.428; unadjusted analyses.
Preziosa et al., 2024 [20]	Normalized CPV	Automated in-house T1/FLAIR method with visual inspection/correction; normalized volumes	BRB-N: verbal/visual memory, SDMT, PASAT, verbal fluency	Abnormal performance in ≥2 cognitive domains = CI	MS had higher nCPV than HCs (2.59 vs. 2.18 mL; *p* = 0.001). CI patients had higher nCPV than cognitively preserved patients (2.82 vs. 2.51 mL; pFDR = 0.009); nCPV predictor importance was 80.3%.	Not assessed
X. Wang et al., 2024 [21]	nCPV (CPV/TIV)	Manual ITK-SNAP masks as ground truth; 3D UX-Net automated segmentation	SDMT	Cognitive worsening = raw SDMT decrease ≥ 4 points or ≥10%	Baseline nCPV was inversely associated with SDMT performance.	Baseline nCPV did not predict 2-y cognitive worsening; nCPV change did not differ by cognitive-worsening status.
De Meo et al., 2025 [22]	nCPV and CP T1/T2 ratio	FreeSurfer v7.4.1 longitudinal pipeline after lesion filling; Gaussian-mixture refinement, visual review, and manual editing; nCPV = CPV/TIV	BICAMS: SDMT, CVLT-II, BVMT-R	Continuous baseline and longitudinal scores; no single binary CI definition	Higher nCPV was independently associated with lower BVMT-R (standardized beta = −0.16, 95% CI −0.27 to −0.05; *p* = 0.004), but not with SDMT or CVLT-II.	Baseline nCPV did not predict cognitive change. Higher CP T1/T2 ratio predicted faster BVMT-R decline (standardized beta = −0.23, 95% CI −0.43 to −0.03; FDR-corrected *p* = 0.04).
Z. Wang et al., 2025 [23]	CPV/TIV × 10^−3^	Manual segmentation with U-Net cross-check	BRB-N: SRT, SDMT, PASAT, WLG, SPART, and delayed recall variants	≥1 abnormal test in ≥2 domains = Co-I	Co-I occurred in 42.9% of RRMS. Co-I patients had higher CPV and lower DTI-ALPS. DTI-ALPS mediated 27.21% of the CPV-SDMT association and 43.75% of the CPV-delayed visuospatial-memory association.	Not assessed
Galus et al., 2026 [24]	Left and right CP volumes normalized to eTIV	FreeSurfer v7.4.1	CVLT, BVRT, CTT, VFT, VST, SDMT	Preserved, single-domain, and multidomain groups based on abnormal-domain count	CI occurred in 59% of RRMS. CP volumes were slightly higher in multidomain CI and negatively correlated with VFT Total and VFT Verb Fluency.	Not assessed

Abbreviations: BICAMS = Brief International Cognitive Assessment for Multiple Sclerosis; BRB-N = Brief Repeatable Battery of Neuropsychological Tests; BVMT-R = Brief Visuospatial Memory Test-Revised; CI = cognitive impairment; Co-I = cognitively impaired; CP = choroid plexus; CPV = choroid plexus volume; CVLT-II = California Verbal Learning Test, Second Edition; DTI-ALPS = diffusion tensor imaging along perivascular spaces; eTIV/TIV = estimated/total intracranial volume; HC = healthy control; IRL = iron rim lesion; MoCA = Montreal Cognitive Assessment; nCPV = normalized CPV; PASAT = Paced Auditory Serial Addition Test; RRMS = relapsing-remitting MS; SDMT = Symbol Digit Modalities Test; SPART = Spatial Recall Test; VFT = Verbal Fluency Test; WCT = Word Comprehension Test.

## Data Availability

All data were extracted from published articles. Eligibility criteria and search strategies are provided as Supplementary Materials.

## Supplementary Materials

The following supporting information can be downloaded at: https://www.mdpi.com/article/10.3390/brainsci16080829/s1, Table S1: Population–Concept–Context framework and eligibility criteria; Table S2. Full electronic database search strategies; Table S3: MRI acquisition and choroid plexus assessment characteristics of the included studies; Table S4: Statistical analysis and methodological appraisal of the included studies.

## Abbreviations

The following abbreviations are used in this manuscript:

BICAMS	Brief International Cognitive Assessment for Multiple Sclerosis
BRB-N	Brief Repeatable Battery of Neuropsychological Tests
BVMT-R	Brief Visuospatial Memory Test–Revised
CI	cognitive impairment
CNS	central nervous system
CP	choroid plexus
CPV	choroid plexus volume
CSF	cerebrospinal fluid
CVLT-II	California Verbal Learning Test, Second Edition
DMT	disease-modifying therapy
DTI-ALPS	diffusion tensor imaging along perivascular spaces
EDSS	Expanded Disability Status Scale
IRL	iron rim lesion
MRI	magnetic resonance imaging
MS	multiple sclerosis
nCPV	normalized choroid plexus volume
PMS	progressive multiple sclerosis
PPMS	primary progressive multiple sclerosis;
PRL	paramagnetic rim lesion
RRMS	relapsing-remitting multiple sclerosis
SDMT	Symbol Digit Modalities Test
TIV/eTIV	total/estimated total intracranial volume
