# Supplementary material for: Choroid Plexus MRI Features and Cognitive Outcomes in Multiple Sclerosis: A Scoping Review
Source: Brain Sci. 2026 Aug 4;16(8):829. doi: 10.3390/brainsci16080829 (PMC13510425; doi:10.3390/brainsci16080829)
Supplement: Supplementary file 1 [file brainsci-16-00829-s001.zip › Supplementary_Material_Table_S1.pdf]

Supplementary Table S1. Population-Concept-Context framework and eligibility criteria.

PCC element	Inclusion criteria	Exclusion criteria	Notes for screening
Population	Adults with clinically diagnosed MS, including all MS phenotypes and care settings.	Pediatric populations; participants with other neurological or demyelinating diseases unless MS data are reported separately; animal or in vitro studies.	Confirm adult human MS population during title/abstract and full-text screening.
Concept 1: choroid plexus	Choroid plexus-specific features, including volume, enlargement, morphology, MRI-derived metrics, segmentation, or other measures specific to the choroid plexus.	Studies focused only on ventricles, cerebrospinal fluid, or other brain structures without separate choroid plexus data.	Record imaging modality, sequence, segmentation method, and whether the measure is absolute or normalized.
Concept 2: cognition	Cognitive functions, cognitive impairment, neuropsychological assessment, or cognitive outcomes in people with MS.	Studies without any cognitive outcome or cognitive assessment; studies focused only on non-cognitive outcomes such as relapse, motor disability, or fatigue without cognition.	Cognition is not required in the database search block but is required for inclusion.
Evidence type	Primary empirical human studies, including cross-sectional, longitudinal, cohort, case-control, observational, and interventional studies.	Reviews, meta-analyses, protocols, editorials, letters, case reports, theoretical articles, and conference abstracts without a full text.	Reviews may be used for citation chaining but not included in the evidence synthesis.
