# Supplementary material for: Choroid Plexus MRI Features and Cognitive Outcomes in Multiple Sclerosis: A Scoping Review
Source: Brain Sci. 2026 Aug 4;16(8):829. doi: 10.3390/brainsci16080829 (PMC13510425; doi:10.3390/brainsci16080829)
Supplement: Supplementary file 1 [file brainsci-16-00829-s001.zip › Supplementary_Material_Table_S2.pdf]

Supplementary Table S2. Full electronic database search strategies.

Database	Search fields / limits	Search strategy	Records retrieved, n
**PubMed**	MeSH terms and title/abstract terms. Animal-only records excluded using NOT (animals[MeSH Terms] NOT humans[MeSH Terms]). No age, language, or study-design filters.	(<br>"Multiple Sclerosis"[MeSH Terms]<br>OR "multiple sclerosis"[tiab]<br>OR "relapsing-remitting multiple sclerosis"[tiab]<br>OR "secondary progressive multiple sclerosis"[tiab]<br>OR "primary progressive multiple sclerosis"[tiab]<br>OR RRMS[tiab]<br>OR SPMS[tiab]<br>OR PPMS[tiab]<br>)<br>AND<br>(<br>"Choroid Plexus"[MeSH Terms]<br>OR "choroid plexus"[tiab]<br>OR "choroid plexuses"[tiab]<br>)<br>NOT (animals[MeSH Terms] NOT humans[MeSH Terms])	203
**Scopus**	TITLE-ABS-KEY fields. No age, language, or study-design filters.	TITLE-ABS-KEY(<br>"multiple sclerosis"<br>OR "relapsing-remitting multiple sclerosis"<br>OR "secondary progressive multiple sclerosis"<br>OR "primary progressive multiple sclerosis"<br>OR RRMS OR SPMS OR PPMS<br>)<br>AND<br>TITLE-ABS-KEY(<br>"choroid plexus"<br>OR "choroid plexuses"<br>)	407
**Web of Science Core Collection**	Topic search (TS). No age, language, or study-design filters.	TS=(<br>"multiple sclerosis"<br>OR "relapsing-remitting multiple sclerosis"<br>OR "secondary progressive multiple sclerosis"<br>OR "primary progressive multiple sclerosis"<br>OR RRMS OR SPMS OR PPMS<br>)<br>AND<br>TS=(<br>"choroid plexus"<br>OR "choroid plexuses"<br>)	550
**Cochrane Library**	Advanced Search / Search Manager. MeSH descriptors and title/abstract/keyword fields. No age, language, or study-design filters.	#1 MeSH descriptor: [Multiple Sclerosis] explode all trees<br>#2 ("multiple sclerosis" OR "relapsing-remitting multiple sclerosis" OR "secondary progressive multiple sclerosis" OR "primary progressive multiple sclerosis" OR RRMS OR SPMS OR PPMS):ti,ab,kw<br>#3 #1 OR #2<br>#4 MeSH descriptor: [Choroid Plexus] explode all trees<br>#5 ("choroid plexus" OR "choroid plexuses"):ti,ab,kw<br>#6 #4 OR #5<br>#7 #3 AND #6	3

All databases were searched during April–May 2026 from inception to the date of each search, without publication-date, language, age, or study-design restrictions.
