# Supplementary material for: Choroid Plexus MRI Features and Cognitive Outcomes in Multiple Sclerosis: A Scoping Review
Source: Brain Sci. 2026 Aug 4;16(8):829. doi: 10.3390/brainsci16080829 (PMC13510425; doi:10.3390/brainsci16080829)
Supplement: Supplementary file 1 [file brainsci-16-00829-s001.zip › Supplementary_Material_Table_S3.pdf]

Supplementary Table S3. MRI acquisition and choroid plexus assessment characteristics of the included studies.

Part A. MRI acquisition characteristics.

Study	Scanner, field strength, and head coil	MRI sequences	Spatial resolution / slice thickness	Main acquisition parameters	Contrast administration and other acquisition notes
X. Wang et al., 2023	Siemens Magnetom Skyra; 3.0 T; 32-channel head coil.	Sagittal 3D T1-weighted MPRAGE (pre- and post-gadolinium); sagittal 3D FLAIR; multi-echo QSM.	T1: 1.0 x 1.0 x 1.0 mm.<br>FLAIR: 0.5 x 0.5 x 1.0 mm.<br>QSM: 0.5 x 0.5 x 2.0 mm.	T1: TE/TR = 2.26/2300 ms; TI = 900 ms; FOV = 256 mm; FA NR.<br>FLAIR: TE/TR = 388/5000 ms; TI = 1800 ms; FOV = 256 mm.<br>QSM: TE = 7.5/15/22.5/30 ms; TR = 36 ms; FOV = 220 mm; FA = 20 degrees.	Pre- and post-gadolinium T1 acquired. QSM reconstructed with STI Suite v3.03.
Kania et al., 2024	Baseline: GE MR750; 3.0 T; 32-channel dedicated head coil.<br>Annual follow-up: Siemens Magnetom Skyra; 3.0 T; 20-channel dedicated head coil.	Baseline: high-resolution T1-weighted BRAVO; CUBE T2-FLAIR; multidirectional T2-weighted diffusion acquisition.<br>Follow-up: annual clinical MRI; sequence details NR.	NR for all sequences.	TR, TE, TI, FA, FOV, matrix, and acquisition time NR.	Annual follow-up MRI recorded new/enlarging T2 and gadolinium-enhancing lesions. CP assessment used baseline MRI.
Preziosa et al., 2024	Philips Ingenia CX; 3.0 T; dS-Head-32 receiving coil.	Sagittal 3D FLAIR; sagittal 3D T1-weighted turbo field echo; 3D T2-weighted; 3D SWI.	FLAIR: 1 x 1 x 1 mm; 192 slices.<br>T1: 1 x 1 x 1 mm; 204 slices.<br>T2: 1 x 1 mm in-plane; 1-mm slice; 192 slices.<br>SWI: 0.60 x 0.60 mm in-plane; 2-mm slice; 135 slices.	FLAIR: TR/TE/TI = 4800/270/1650 ms; ETL = 167.<br>T1: TR/TE/TI = 7/3.2/1000 ms; FA = 8 degrees.<br>T2: TR/TE = 2500/330 ms; ETL = 117.<br>SWI: TR = 39 ms; TEs = 5.5:6:35.5 ms; FA = 17 degrees.	Contrast administration NR. Slices oriented parallel to a line joining the infero-anterior and infero-posterior margins of the corpus callosum.

Study	Scanner, field strength, and head coil	MRI sequences	Spatial resolution / slice thickness	Main acquisition parameters	Contrast administration and other acquisition notes
X. Wang et al., 2024	Internal datasets: Siemens Magnetom Skyra; 3.0 T; 32-channel head coil. External dataset: GE Discovery MR750, Siemens Skyra, Siemens TrioTim, and GE Healthcare scanner; field strengths and coils NR.	Internal: sagittal 3D T1-weighted MPRAGE. External: 3D T1-weighted imaging acquired with non-standardized clinical protocols.	Internal and all external sites: 1.0 x 1.0 x 1.0 mm.	Internal: TE = 2.3 ms; TR = 2300 ms; TI = 900 ms; FA = 8 degrees; matrix = 256 x 256 x 192. External site 1: TR/TE = 8.2/3.2 ms; TI = 450 ms; FA = 12 degrees; matrix = 256 x 256 x 196. Site 2: 1900/2.3 ms; TI = 900 ms; FA = 9 degrees; 256 x 256 x 176. Site 3: 1600/2.1 ms; TI = 1000 ms; FA = 9 degrees; 256 x 224 x 176. Site 4: 8.6/3.4 ms; TI = 400 ms; FA = 12 degrees; 256 x 256 x 156.	Contrast administration NR. External protocols were explicitly non-standardized.
Z. Wang et al., 2025	Philips Ingenia CX; 3.0 T; 32-channel phased-array head coil.	Sagittal 3D T1-weighted MPRAGE; sagittal 3D FLAIR; single-shot EPI diffusion MRI (64 directions; b = 0 and 1000 s/mm2).	T1: 1 x 1 x 1 mm; 107 slices; matrix = 256 x 256. FLAIR: 1 x 1 x 1 mm; 260 slices; matrix = 252 x 251. Diffusion: 2 x 2 x 2 mm; 70 slices; matrix = 112 x 109.	T1: TR/TE = 6.4/2.9 ms; FA = 8 degrees; FOV = 256 x 256 mm. FLAIR: TR/TE = 4800/340 ms; FA = 180 degrees; FOV = 250 x 250 mm. Diffusion: TR/TE = 8000/85 ms; FOV = 224 x 224 mm.	Contrast administration NR. MRI was performed within 48 h of cognitive assessment and between 8 pm and 10 pm.
De Meo et al., 2025	Philips Ingenia CX; 3.0 T; head coil NR.	3D T1-weighted GRE; 2D T2-weighted turbo spin echo; 3D FLAIR.	T1: 1 x 1 x 1 mm. T2: 1 x 1 x 3 mm. FLAIR: 1 x 1 x 1 mm.	T1: TR/TE = 6.9/3.1 ms; FA = 8 degrees. T2: TR = 3500 ms; TE = 8/19 ms; turbo factor = 10. FLAIR: TR/TE/TI = 8000/388/2400 ms; turbo factor = 120.	Contrast administration NR. Same protocol at baseline, 6 months, and 18 months. Slices positioned parallel to a line joining the infero-anterior and infero-posterior corpus callosum margins.
Galus et al., 2026	GE SIGNA Artist; 1.5 T; 19-channel Head & Neck coil.	Axial precontrast T1-weighted MP-RAGE; sagittal T2-weighted FLAIR FS CUBE.	NR.	TR, TE, TI, FA, FOV, matrix, slice number, and acquisition time NR.	Head MRI with contrast was obtained when possible; only the precontrast T1 volumetric series was used for volumetry. Images were filtered with SCENIC and stored in DICOM format.

Part B. Choroid plexus assessment characteristics.

Study	CP compartment and input images	Segmentation method and software	Preprocessing / quality control	Normalization and CP metric	Validation / reliability
X. Wang et al., 2023	CP of the lateral ventricles on 3D T1-MPRAGE. CP in the third and fourth ventricles was excluded.	Manual segmentation in ITK-SNAP v3.8 in axial, sagittal, and coronal planes by a junior neuroradiologist; reviewed and corrected by a senior neuroradiologist.	T1-MPRAGE used for CP delineation; no CP-specific automated preprocessing described.	CPV divided by TIV (CPV/TIV).	Expert review and correction reported; numerical inter- or intra-rater reliability NR.
Kania et al., 2024	Left and right CP compartments derived from baseline high-resolution T1-weighted MRI.	FreeSurfer v5.3 default recon-all pipeline; compartment volumes extracted with asegstats2table.	DICOM-to-NIfTI conversion; standard FreeSurfer intensity correction, registration, skull stripping, and atlas-based labeling. Technically acceptable baseline MRI available for 27/30 participants.	Left and right CP volumes were analyzed; CP-specific normalization NR. FreeSurfer-estimated ICV was available but was not stated as the CP denominator.	No CP-specific manual correction or numerical segmentation reliability reported.
Preziosa et al., 2024	Lateral-ventricular CP; 3D T1-weighted and 3D FLAIR images used as inputs.	Fully automatic in-house method starting from FSL-SIENAX tissue segmentation; implemented in MATLAB R2017a. Registered MNI-152 lateral ventricle mask, two-stage Gaussian mixture modeling, and cluster filtering.	Lateral ventricle mask affinely registered and eroded by 1 mm; WM, GM, and lesion masks subtracted; mean filtering; residual clusters <3 mm removed. All masks visually inspected and manually corrected if inaccurate.	Reported as normalized CP volume (mL); the article did not separately specify the CP normalization formula.	Visual inspection and manual correction of all segmentations; numerical CP reliability metric NR.
X. Wang et al., 2024	Lateral-ventricular CP on 3D T1-weighted images. CP in the third and fourth ventricles was excluded.	Manual ITK-SNAP v3.8 masks served as ground truth. Automatic segmentation used 3D UX-Net; 3D U-Net was the comparator.	N4 bias-field correction; reorientation; resampling to isotropic 1-mm voxels; cropping/padding to 182 x 218 x 182; Z-score intensity normalization. Manual labels reviewed and corrected by a senior neuroradiologist.	nCPV = CPV/TIV x 10^3; TIV obtained with FreeSurfer.	3D UX-Net Dice: 0.879 (validation), 0.875 (internal test), and 0.870 (external test). Automatic vs manual CPV correlation: r = 0.921 internally and r = 0.807 externally; Bland-Altman analysis performed.

Study	CP compartment and input images	Segmentation method and software	Preprocessing / quality control	Normalization and CP metric	Validation / reliability
Z. Wang et al., 2025	CP manually delineated on coronal 3D T1-weighted images; exact ventricular compartment not explicitly stated.	Manual segmentation in ITK-SNAP v3.6 by two blinded neuroradiologists; uncertainties reviewed and corrected by a senior radiologist. U-Net segmentation used as a cross-check.	Automatic cross-check included nonlinear registration to the ICBM-MNI152 T1 template. Manual uncertainty review used sagittal and axial planes.	Normalized CPV = raw CPV/TIV, reported in units of 10^-3.	Inter-observer ICC = 0.875 (95% CI 0.824-0.912); intra-observer ICC = 0.948 (0.911-0.970); manual vs U-Net ICC = 0.882 (0.833-0.917).
De Meo et al., 2025	Left and right lateral-ventricular CP on 3D T1-weighted images; bilateral values were summed because no side difference was observed.	FreeSurfer v7.4.1 longitudinal pipeline followed by Bayesian Gaussian mixture model refinement. First two-component GMM within the lateral ventricle mask; 3D SUSAN smoothing (sigma = 1 mm); second three-component GMM. Experienced observer reviewed and manually edited masks when needed.	T1-hypointense lesion filling before FreeSurfer. For CP T1/T2 maps: rigid T2-to-T1 registration with FLIRT, N4 bias correction with ANTs, nonlinear intensity normalization, and one-voxel mask erosion to reduce partial-volume effects.	NChPV = CPV/TIV. Additional CP microstructural metric: mean CP T1/T2 ratio.	Experienced visual review and manual editing reported; numerical CP segmentation reliability NR.
Galus et al., 2026	Left and right CP compartments from axial precontrast T1-weighted MP-RAGE.	Automated FreeSurfer v7.4.1 regional segmentation.	SCENIC filtering before volumetry. No CP-specific manual correction or dedicated CP quality-control procedure reported.	Left and right CP volumes normalized to eTIV, consistent with the study-wide regional volume normalization procedure.	CP-specific validation or numerical reliability NR.

Abbreviations: 2D, two-dimensional; 3D, three-dimensional; ANTs, Advanced Normalization Tools; CP, choroid plexus; CPV, choroid plexus volume; eTIV/TIV, estimated/total intracranial volume; ETL, echo train length; FA, flip angle; FLAIR, fluid-attenuated inversion recovery; FOV, field of view; Gd, gadolinium; GMM, Gaussian mixture model; GRE, gradient echo; ICC, intraclass correlation coefficient; ICV, intracranial volume; MPRAGE/MP-RAGE, magnetization-prepared rapid gradient echo; NR, not reported; QSM, quantitative susceptibility mapping; SWI, susceptibility-weighted imaging; TE, echo time; TI, inversion time; TR, repetition time.
