# Supplementary material for: Choroid Plexus MRI Features and Cognitive Outcomes in Multiple Sclerosis: A Scoping Review
Source: Brain Sci. 2026 Aug 4;16(8):829. doi: 10.3390/brainsci16080829 (PMC13510425; doi:10.3390/brainsci16080829)
Supplement: Supplementary file 1 [file brainsci-16-00829-s001.zip › Supplementary_Material_Table_S4.pdf]

Supplementary Table S4. Statistical analysis and methodological appraisal of the included studies.

Study	Statistical model / effect estimate	Covariates	Multiple-comparison correction	Reliability / validation	Main methodological limitations	JBI tool / overall appraisal
X. Wang et al., 2023 [18]	Group tests, logistic regression, Pearson correlations, and multivariable regression. CPV: RRMS vs HCs +30% ($p < 0.001$); IRL-positive vs IRL-negative +20% ($p = 0.007$); CPV-WML $r = 0.46$; CPV-IRL $r = 0.51$; CI-group difference $p = 0.011$. SDMT association was attenuated after adjustment.	Lesion load and brain volumes in adjusted cognition/EDSS models	NR	Manual ITK-SNAP segmentation; CP-specific inter-/intra-rater reliability NR	Cross-sectional; RRMS only; only lateral-ventricle CP assessed; MoCA is a screening measure; adjusted CP-cognition associations were non-significant	JBI analytical cross-sectional / Some concerns
Kania et al., 2024 [19]	Wilcoxon signed-rank tests and Spearman correlations. CP vs follow-up semantic fluency: $r = -0.068$ and 0.103; CP vs WCT: $r = -0.091$ and -0.159; all $p >= 0.428$.	None in CP-cognition analyses	None reported	FreeSurfer v5.3; CP-specific normalization and reliability NR	Small cohort and attrition (41 to 30; MRI n = 27); no control group; no adjustment for baseline cognition or major confounders; no follow-up volumetry; treatment switching and possible practice effects	JBI cohort / Some concerns
Preziosa et al., 2024 [20]	Group comparisons; negative-binomial, linear, logistic, and quasibinomial models; Boruta random forest. CI vs cognitively preserved nCPV: 2.82 vs 2.51 mL ($p_{FDR} = 0.009$); predictor importance 80.3%.	Age and sex; CP models additionally adjusted for normalized brain and lateral-ventricle volumes	FDR correction	Automated in-house segmentation with visual inspection/correction; formal CP reliability metrics NR in the review table	Cross-sectional; mixed MS phenotypes; no T1 spin-echo black-hole assessment	JBI analytical cross-sectional / Low concerns
X. Wang et al., 2024 [21]	Partial correlations, logistic regression, sign tests, and GLMs. Baseline nCPV was inversely associated with SDMT but did not predict 2-y cognitive worsening. 3D UX-Net Dice: 0.875 internal and 0.870 external.	Age, sex, education, and disease duration; longitudinal models also included baseline nCPV	NR	Manual ground truth with internal and external 3D UX-Net validation	Study primarily validated segmentation; cognition limited to SDMT; external dataset lacked SDMT; limited follow-up events; RRMS only; limited DMT subgroup	JBI cohort checklist, adapted / Some concerns

Study	Statistical model / effect estimate	Covariates	Multiple-comparison correction	Reliability / validation	Main methodological limitations	JBI tool / overall appraisal
De Meo et al., 2025 [22]	Multiple linear regression, mixed-effects models, and ACE trajectory modelling. nCPV-BVMT-R: beta = -0.16 (95% CI -0.27 to -0.05; p = 0.004). CP T1/T2-BVMT-R decline: beta = -0.23 (95% CI -0.43 to -0.03; pFDR = 0.04).	Age, gender, disease duration, DMT class, and baseline CP metrics where relevant; lateral-ventricle sensitivity analyses	Benjamini-Hochberg FDR, except stepwise selection	FreeSurfer longitudinal pipeline, lesion filling, Gaussian-mixture refinement, visual review, and manual editing	RRMS only; attrition (276/422 at 6 mo; 80/422 at 18 mo); short/variable follow-up; treatment-initiation and pseudoatrophy effects; possible practice effects; no healthy controls	JBI cohort / Some concerns
Z. Wang et al., 2025 [23]	Multivariable regression, PROCESS mediation, and Boruta random forest. DTI-ALPS mediated 27.21% of CPV-SDMT and 43.75% of CPV-delayed visuospatial-memory associations.	Regression: sex, age, and DMT; mediation: age, sex, and education	FDR correction	Manual CP segmentation with inter-/intra-rater assessment and U-Net cross-check; numeric reliability values NR in the review table	Cross-sectional; relatively small sample; RRMS only; ROI-based DTI-ALPS may not represent whole-brain glymphatic function; no gray-matter microstructural analysis	JBI analytical cross-sectional / Low concerns
Galus et al., 2026 [24]	Group comparisons, Pearson correlations, and partial correlations. CP volumes were slightly higher in multidomain CI and negatively correlated with VFT Total and VFT Verb Fluency.	Age, education >12 y, and BDI-II in partial correlations	NR for CP-cognition analyses	FreeSurfer v7.4.1; CP-specific validation/reliability NR	Small sample, especially PPMS and cognitive subgroups; cross-sectional; some international norms; no DTI/fMRI; limited adjustment for lesion burden, ventricular volume, global atrophy, EDSS, and disease duration	JBI analytical cross-sectional / Some concerns

Abbreviations: ACE = alternating conditional expectation; BDI-II = Beck Depression Inventory-II; CI = cognitive impairment; CP = choroid plexus; CPV = choroid plexus volume; DMT = disease-modifying therapy; DTI-ALPS = diffusion tensor imaging along perivascular spaces; FDR = false discovery rate; HC = healthy control; IRL = iron rim lesion; JBI = Joanna Briggs Institute; NR = not reported; RRMS = relapsing-remitting MS; SDMT = Symbol Digit Modalities Test; VFT = Verbal Fluency Test; WCT = Word Comprehension Test; WML = white matter lesion.
